# Does Public Health Emergency Experience Have an Impact on Individual Reactions during COVID-19?

**DOI:** 10.3390/healthcare11091212

**Published:** 2023-04-24

**Authors:** Chenggang Zhang, Pengrui Ou, Pengfei Guo

**Affiliations:** 1 Department of Sociology, Tsinghua University, Beijing 100084, China; opr21@mails.tsinghua.edu.cn; 2 Department of Environmental Health Sciences, Yale School of Public Health, Yale University, New Haven, CT 06520, USA; pengfei.guo@yale.edu

**Keywords:** life course, risk study, public health emergency, COVID-19

## Abstract

Local historical experience in public health emergencies has been perceived to largely affect COVID-19’s social influence. Specifically, individuals’ personal experience in public health emergencies would likely have an impact on their reactions to the next similar event. Herein, we combined life course and risk analysis frameworks to explore how individuals’ experiences influence current risk perception and protective behaviors. We collected 1000 questionnaires of random network samples in six Chinese provinces of different risk levels from 29 April to 8 May 2020, and used the propensity score matching (PSM) model and multivariable linear regression to process the data. We categorized individual public emergency experience into three patterns: (1) having ever witnessed a public health emergency, (2) having ever experienced a public health emergency, and (3) currently experiencing a public health emergency. The study indicates that individuals’ experiences had significant positive effects on protective behaviors against COVID-19. The average effects of the three patterns on behaviors were 0.371 (*p* < 0.001), 0.898 (*p* < 0.001) and 0.319 (*p* < 0.05), respectively. The study also shows that for those experiencing any one pattern, the effect of risk perception on protective behaviors appeared null in the early stage of the pandemic. We propose the potential interactive mechanism of risk factors in the life course at the individual level. Academically, this study develops the risk theory of perception and behavior and expands the application of the life course approach in the public health arena. Practically, our research indicates that public health emergency experiences are valuable for responding to a future pandemic and normalizing prevention policies.

## 1. Introduction

It has been three years since the World Health Organization (WHO) declared COVID-19 a public health emergency of international concern (PHEIC) in January 2020. Globally, by 7 January 2023, there had been more than 667 million confirmed COVID-19 cases, including 6.7 million deaths, reported by the WHO [1]. Notably, the number of cumulative total cases in the western Pacific and Southeast Asia was 169 million, which is far lower than that in low-density regions such as Europe and the Americas, at 457 million [2]. Most of the Asian regions experienced a similar pandemic—SARS—in the early 21st century [3], with comparable contagiousness and deadliness to COVID-19. In contrast, the most recent similar threatening pandemic for Western countries can be traced back to the 1918 influenza. Therefore, we hypothesize that personal experience of public health emergencies may affect the level of individual risk perception and protective behaviors during COVID-19.

In this study, risk perception refers to the subjective judgment about the characteristics and severity of a risk. Risk perception includes the perception of risk familiarity, controllability, and fear, which have been considered a motivation for protective behaviors [4]. Studies on COVID-19 around the world have found that risk perception is highly associated with protective behavior practices, such as social distancing, cough etiquette, and handwashing (hand hygiene) [5,6]. For example, a recent cross-sectional study among UK college students identified risk perception, personal habits, and the passage of time as the most important predictors of protective behaviors [7]. A China-based study also found a strong correlation between risk perception and the level of risk prevention behavior, and the correlation magnitude was different by sex, age, and education [8]. However, many factors, including personal experience of public health emergencies, may affect both risk perception and protective behaviors, and thus if not adjusted, could confound the relationship between risk perception and protective behaviors.

To disentangle the relationship between personal experience, risk perception, and protective behaviors, there exists a potential mechanism that risk perception and protective behaviors during a public health emergency may depend on personal life trajectory. Vickerstaff attempted to combine the frameworks of the life course approach and risk analysis, and found that risk was merely presented as factors affecting the life course, as a collection of sociopolitical, economic, cultural, natural, and other uncertain events [9]. Risk was at a population level and life course at an individual level in her research, without discussing how risk is constructed, perceived, or protected against at an individual level.

Not only Vickerstaff’s research but also most of the relevant studies do not integrate these two frameworks at an individual level [9]. In terms of their theoretical orientation, use of concepts, and analytical frameworks, these works can be broadly classified into “life course research involving risk elements” and “risk research involving life course elements.” In the first category, most studies consider perceived risk as a psychological factor, like the concept of self-efficacy, and treat it as an independent variable. They studied the relationship between individual life experience and risk response without placing the variable, risk, in the context of the life course [8,10,11,12]. Moreover, in the category of “risk research involving life course elements”, the focus has largely been on elements related to life course and trajectory, but the reasons that differences in age-groups cause differences in risk perceptions and choices and the real latent variables behind the independent variable of age-group have generally been underexplored in risk research.

Taken together, life course studies and risk studies share a wide variety of common variables in relevant research in public health, which differ in expression but can interlink when operationalized, such as age [13,14,15], occupation [16,17], location, income [18], social connections [19], individual experience [20], and collective experience [21,22]. Despite the insufficient exploration of the theoretical combination, the variables involved in all these studies above offer the possibility of merging the two frameworks.

In this study, we aimed to examine:

(1) the impact of public health emergency experience on risk perception and protective behaviors;
(2) the association between risk perception and protective behavior conditioning on public health emergency experience;
(3) the moderating role of public health emergency experience on the relationship between risk perception and protective behaviors.

To sum up, we integrate individuals’ lives with their whole social history and regard public health emergencies as a part of people’s life course, which will help to understand one’s risk perception and behavior in a new way, and explore the implications of the findings for the theoretical understanding of the relationship between life course and risk theory. This study not only has theoretical significance in the realms of life course theory and risk theory but also provides practical approaches to deal with normalized, repeated risks in modern society.

## 2. Methods

### 2.1. Study Design and Participants

The earliest COVID-19 cases in China were from Wuhan city, Hubei province in November 2019. This study adopted the method of an online questionnaire and entrusted the data collection to the professional survey organization Love Research Company, which randomly distributed the questionnaire links to qualifying participants in the Love Research community (sample library). Implementation started on 29 April 2020 and ended on 8 May 2020. The survey finally recycled 1000 valid questionnaires, for which the response rate was 18% and the average completion time 13 min.

In terms of the selection of target groups, the survey made the following quotas. Firstly, at the regional level, the survey divided the country into high-risk areas (more than 10,000 confirmed cases), medium-risk areas (1000–10,000 confirmed cases), and low-risk areas (less than 1000 confirmed cases) according to the cumulative number of confirmed cases in the country on 25 April 2020. Six provinces—Hubei (500 cases), Zhejiang (100 cases), Guangdong (100 cases) Henan (100), Shanxi (100), and Guizhou (100) were selected as the target provinces—among which, except Hubei Province, other provinces adopted the method of random sampling. On 25 April 2020, Hubei had 68,128 confirmed cases, making it a high-risk province. Zhejiang, Guangdong, and Henan had 1268, 1585, and 1276 confirmed cases, respectively, making them medium-risk provinces. Shanxi and Guizhou, with 197 and 147 confirmed cases respectively, were low-risk provinces. Therefore, we included Hubei (*n* = 500), Zhejiang (*n* = 100), Guangdong (*n* = 100), Henan (*n* = 100), Shanxi (*n* = 100), and Guizhou (*n* = 100)). For Hubei, according to the risk level and urban population of each city, Wuhan (225), Xiaogan (110), Huanggang (140) and Qianjiang (25) were selected as the target cities. To ensure the representativeness of the sample, this study controls the sex ratio, urban-rural ratio, and age structure of the sample based on the results of the sixth national census). Secondly, at the sex level, sampling was conducted on a 1:1 basis. Thirdly, at the age level, according to the results of the sixth national population census, approximately 260 samples aged 18–30, 200 samples aged 31–40, 220 samples aged 41–50, 150 samples aged 51–60, and 170 samples aged over 61 were selected. Lastly, at the level of urban and rural distribution, samples were taken at 6:4 in urban and rural areas where the epidemic occurred.

The above sampling methods take full account of factors such as region, age, sex, and urban and rural areas, and are comprehensive to some extent, but also have some limitations. For example, the sampling at the regional level does not follow the unified standard. Although taking into account the reality of the “severely affected areas” of Hubei, it cannot guarantee the representativeness of the samples from other provinces.

The Institutional Review Board of Beijing Normal University approved this study. The participants were told that responses would be anonymous and confidential, and informed consent was obtained from each participant when they received the questionnaires.

### 2.2. Outcomes

The outcomes were risk perception and individual protective behaviors (Table 1). We assessed risk perception on four dimensions: catastrophic potential, familiarity, control, and dread [4,23]. These were scored using Likert scales. Each dimension had a sum of the scores from several items, and we calculated the total score of risk perception by summing up the standardized dimension scores with further standardization on the sum. The individual protective measure was assessed in the question “Do you conduct any of the following measures during the pandemic?” There were 10 measures on the list, for example, washing hands, wearing masks, and reducing the frequency of going outdoors. We calculated the total score of individual protective measures by summing up the number of conducted measures with further standardization on the sum (Table 2).

### 2.3. Exposure Variables

The exposures of interest were life-course risk experiences of public health emergencies. We created three binary exposure variables (yes/no) in our study: (1) having ever witnessed a public health emergency before the COVID-19 pandemic, (2) having ever experienced (directly exposed to) a public health emergency before the COVID-19 pandemic, and (3) currently experiencing a public health emergency.

**Figure 1:**
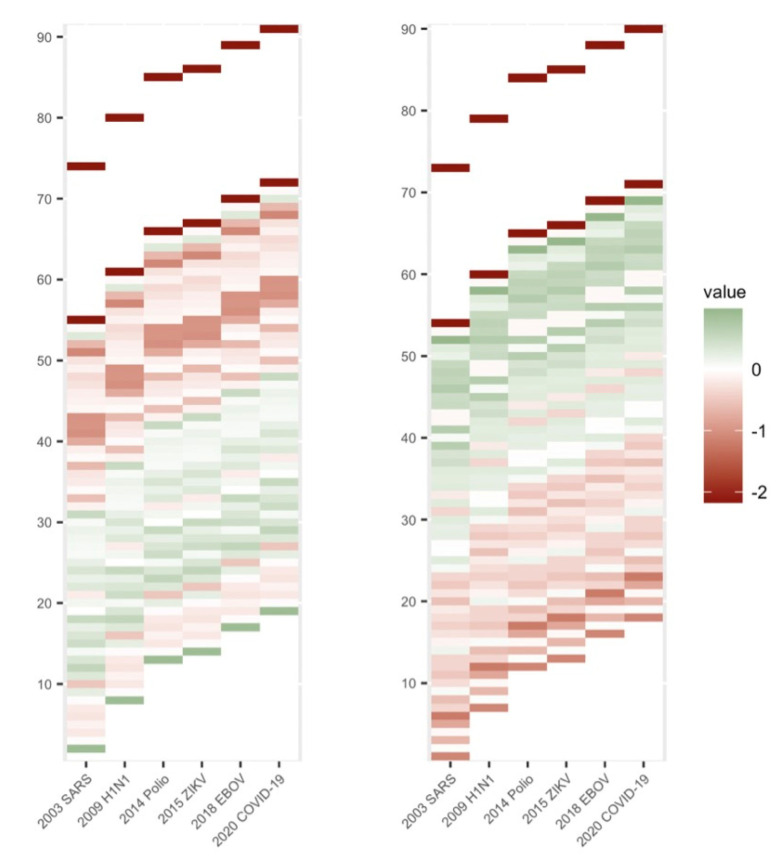
Distribution of risk perception (**left**) and protective measures (**right**) for different ages.

We constructed the first exposure variable based on participant age, personal risk perception, and personal protective measures. Cohort effects are important components in life course research [24]. Riley proposed age-graded life patterns that reflected societal changes [25]. This approach connects cohorts and society structures, categorizes individuals into cohort groups, and evaluates the impact of historical society structures on different cohort groups [25]. Public health emergencies could be considered nodes for cohort group categorization [26]; therefore, we used heat maps to show the distribution of samples for age, risk perception, and protective measures under time nodes of major public health events since 2000. In Figure 1, the stratification of risk perception is not completely clear, but for risk protective measures it is significant. However, in general, there is a breakpoint for both indicators at around 45 years old. The risk perception of samples above the breakpoint is significantly lower than that below the breakpoint, while the level of protective measures is higher than that below the breakpoint, and there is an obvious peer group effect.

In order to determine more specific breakpoints for age-groups, cluster analysis was further used to incorporate sample age, risk perception and protection measures into the model (Table 3). Our model categorized our samples into two cohort groups: one was aged 19–46 (*n* = 620), defined as not having witnessed a public health emergency before the COVID-19 pandemic, and the other was aged 47–91 (*n* = 380), defined as having ever witnessed a public health emergency before COVID-19 pandemic.

The first variable indicated that whether living through the period of any public health emergency, being a witness did not necessarily tell whether individuals were directly exposed to the risk or even ever got infected. Individuals faced directly with public health emergencies might get equipped with a higher level of knowledge, understanding, and protective measures. Therefore, for the second variable, we asked, “Were you directly exposed to any public health emergency as a patient or a frontline worker before the COVID-19 pandemic (e.g., 2002–2004 SARS outbreak, 2009 H1N1 pandemic, 2008–2009 hand, foot, and mouth disease outbreak)?” [27].

The third variable considered current exposure to the COVID-19 pandemic. In risk analysis, occupation is an important indicator of exposed risk level. In infectious disease prevention, medical staff, pathogen researchers, community workers, and volunteers are frontline workers with high-risk exposure. Therefore, we asked, “During this COVID-19 pandemic, what is your role/identity?” If the answer was “medical workers”, “researchers involved in COVID-19”, “heads of COVID-19-related voluntary organizations”, “COVID-19-related government workers”, “community workers involved in COVID-19-related work”, or “producers of COVID-19-related materials”, we coded these participants as “currently experiencing public health emergency.”

### 2.4. Covariates

Based on the literature, covariates included sex, family income, education, marriage status, occupation, and residence. Additionally, for PSM models, we included extra covariates for propensity score matching: family member status and self-evaluation variables. The former included minor family members’ status (<18), elderly family members’ status (>60), and family members’ chronic disease status. The latter included self-evaluation of social status, health, trust in society, and trust in authority. To measure those variables, we asked the following four questions. “What socioeconomic class do you think you belong to?” “What do you think is your current health status?” “How do you personally feel about your level of trust in others (the higher the level, the more likely you are to trust others)?” “How do you personally feel about your level of trust in authority (the higher the level, the more likely you are to trust authority)?” The answers to these questions are given in Table 4.

### 2.5. Statistical Analysis

Our study applied a multivariable linear regression model and propensity score matching (PSM).

Firstly, we introduced PSM to examine the causal effect of public health emergency experience on risk perception and protective behaviors. PSM is designed to facilitate accurate causal inference and good estimation of unobserved potential outcomes by balancing nonequivalent groups in observational studies [28]. PS is the probability of receiving the treatment (or exposure) given the observed covariates [29]. After matching the PS, the PSM model could estimate the average effect of treatment in the treated individuals (ATT) [30]:

$$\begin{array}{cc} \mathrm{ATT} & =E(Y_{i}^{T}-Y_{i}^{C}{|D}_{i}=1)=E\left(Y_{i}^{T}{|D}_{i}=1\right)=E\left(Y_{i}^{C}{|D}_{i}=1\right)\\ & =[E\left(Y_{i}^{T}{|D}_{i}=1\right)-\left.E\left(Y_{i}^{C}{|D}_{i}=0\right)\right]\\ & -\left[E\left(Y_{i}^{C}{|D}_{i}=1\right)-E\left(Y_{i}^{C}{|D}_{i}=0\right)\right]=E(\delta )-\mu \end{array}$$

In the formula, T and C represent the experimental group and the control group, respectively. E(δ) is the difference in observed values between the treated and the untreated, and μ is a sample selection bias that is difficult to observe [29]. In this study, E(δ) denotes the change in risk perception or protective measures. When μ is omittable, E(δ) approximates ATT. If ATT > 0, the treated have a higher level of risk perception or protective measures compared with the untreated, and vice versa. For this study, we estimated ATTs of three exposures on two outcomes in the PSM models.

To guarantee the validity of our results, we conducted a *t*-test for all ATTs and sensitivity analysis for sample selection bias. The sensitivity analysis compared the odds of individuals receiving treatment (π). If individuals have equal odds for entering the treatment group, sample selection bias is minimized. The ratio of odds of entering the treatment group in individual j and individual k could be calculated as follows.

The indicator for sensitivity analysis is Γ. The lower limit of Γ within a 95% confidence interval can be known through the test, and it is generally accepted that if Γ is greater than or equal to 2, the sensitivity test is passed [29,31].

$$\frac{1}{\Gamma }\leq \frac{\pi_{j}\left(1-\pi_{k}\right)}{\pi_{k}\left(1-\pi_{j}\right)}\leq \Gamma \;\mathrm{if}\;j\neq k$$

Considering the stability of PSM methods, we examined various propensity matching methods: k-nearest neighbors match, radius match, kernel match, and Mahalanobis distance match.

Secondly, we used multivariable linear regression to examine the association between risk perception on protective measures adjusting for life-course risk experiences in all participants. We also employed stratified analysis for multivariable linear regression according to the three exposure variables (having ever witnessed, yes/no; having ever experienced, yes/no; currently experiencing, yes/no).

### 2.6. Characteristics of Study Participants

We started with a descriptive statistic. Table 4 shows the characteristics of study participants in this study. Nearly half (47.1%) resided in cities. More than half (63.1%) had completed university or college education. The sex ratio was well balanced in this study sample, with a wide age range in adults and a major group of married people (83.0%).

## 3. Results

### 3.1. PSM Model Results

We used the PSM model to analyze the impact of public health emergency experiences on risk perception and protective behaviors. Personal experience of public health emergencies had largely null effects on risk perception (Table 5). In contrast, personal experience had significant positive effects on protective behaviors against COVID-19, and the results were consistent among different experiences and PSM methods (Table 6). Previous direct exposure to a public health emergency had the largest effect on the level of protective behavior during the present pandemic (ATT = 0.898, *p* < 0.001) compared with the other two types of experiences. In addition, previous experience of public health emergencies as a witness, though not necessarily directly exposed to risks, also significantly affected current protective practices against risks (ATT = 0.371, *p* < 0.001). Current direct exposure to public health emergencies increased protective behavior levels by 0.319 (*p* < 0.05) on average, which was weaker than the effects of previous personal experiences. Therefore, previous personal experience may have a stronger effect on current protective behaviors against risks than current experience.

### 3.2. Linear Regression Model Results

We used linear regression to examine the association between risk perception on protective measures according to personal experience of public health emergencies (Table 7). Overall, a one-point increase in risk perception scores was associated with a 0.179-point increase in protective measure levels on average after adjusting for life-course risk experiences (*p* < 0.001). In stratified analyses, risk perception showed consistently divergent associations with protective measures across different personal experience types. Specifically, for participants who had witnessed, experienced, or were currently experiencing a public health emergency, a one-point increase in risk perception scores was associated with approximately a 0.210-(*p* < 0.001), 0.165-(*p* < 0.001), and 0.206-point (*p* < 0.001) increase in protective measure levels, respectively.

## 4. Discussion

### 4.1. Main Results

According to the analysis of 1000 questionnaires in China, this paper suggests classifying individual experience of public health emergency into three categories: (1) having ever witnessed a public health emergency, (2) having ever experienced a public health emergency, and (3) currently experiencing a public health emergency. Based on the PSM and linear regression model, individual experience has little impact on risk perception, but has a significant impact on protective behaviors. In addition, for the participants who had experience in any one pattern, the effect of risk perception on protective behaviors was no longer significant.

On one hand, risk perception is not only influenced by individual knowledge based on experiences but also by various factors, such as personal values, access to information, and risk communication. Therefore, the weak or null associations between current risk perception and past experiences are probably because of the impact of the latest risk communication and information on perception. As the frequency of social emergencies increases, people are no longer extremely nervous or relaxed during COVID-19. Besides, they can make their own choices effectively concerning the current situation. On the other hand, perception is a relatively well-developed cognitive system after information and behavior construction, rather than an original perception of new emergencies [32,33]. Faced with emerging public health emergencies for which external information is still incomplete and internal cognition is in the process of being constructed, it is conceivable that the knowledge system developed through previous experiences will be more effective in guiding protective behavior.

Slightly different from the classic theory, risk perceptions do not necessarily translate into parallel protective behavior during COVID-19 given the same experience in public health emergencies. This finding is supported in many empirical studies during COVID-19. For example, Lyu et al.’s research on nurses in COVID-19 suggested no clear and direct relationship between risk perceptions and behavioral practices of infection prevention [34]. A significant proportion of those who have been exposed to or suffered a public health emergency may develop a survivor mentality to underestimate the severity and manageability of the risk [35]. This kind of “optimism bias” has been proposed in previous research on H1NI, which would decrease the overall level of protection in society [36]. In addition, for those who are currently experiencing the pandemic, their level of protection could be always high due to their profession. However, because of their occupational requirements, they are invariably exposed to public health risks, hence the vulnerability and insensibility of risk perception.

### 4.2. Interaction Mechanism of Risk Factors in the Life Course

**Figure 2:**
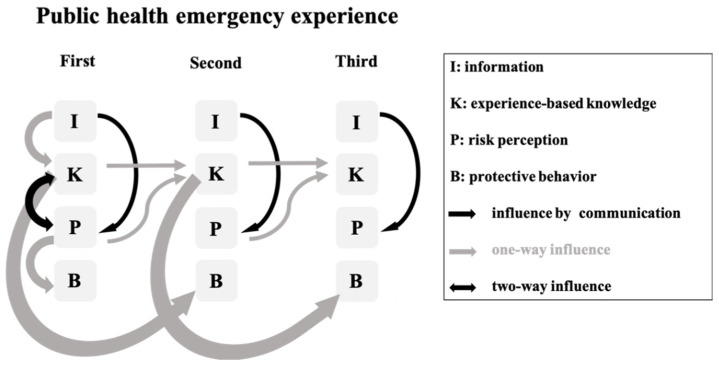
Interrelated external information, individual knowledge, risk perception, and protective measures.

A diagram of the interaction mechanism between individual experience, risk perception, and protective behaviors over the life course is given in Figure 2. Three types of individual experience are used as examples to demonstrate the relationship between the factors: external information, individual knowledge, risk perception, and protective measures during public health emergencies. Of those factors, external information, the highest-level element, is not influenced by the subjective mind, but will affect the information receiver’s risk perception [37,38,39], but behavior, the downstream element, is a manifestation of other factors without affecting risk knowledge or risk perception. In the first individual experience of a major health emergency, an individual’s risk knowledge system is too underdeveloped to affect protective measures, but there exists the inter-construction of knowledge and perception. However, in the next similar experience, stabilized knowledge plays a greater role than perception and influences the risk protection measures.

### 4.3. Theoretical Integration of Public Health Risk and Life Course

Many scholars have attempted the integration of public health risk research and life course theory, and it is important to incorporate a life course perspective into the study of risk in public health. Firstly, public health emergencies are concentrated without requiring immediate action, unlike events such as earthquakes and fires, which require responses in an instant, or environmental risks, which are often belatedly perceived [40]. Therefore, perceptions and behaviors in the early stages of public health emergencies are largely influenced by previous knowledge, which will potentially create both sensitivity to and ignorance of risk [35]. From a historical perspective, people developed general judgments about public health emergencies from many time points, which also led to population differentiation regarding whether and to what extent they have been experienced. Incomprehension of an individual’s past and the situation will make it difficult to determine the causes of one’s risk reactions, that is, only relying on the description of the current phenomenon would not serve as an accurate risk assessment.

In addition, methodologically, most existing research is cross-sectional because of the difficulty in obtaining longitudinal follow-up data and the inaccuracy in respondents’ recall of their past performance at risk [21]. However, the inclusion of a life course framework, combined with the trinity of individual life, sociocultural and historical context, may better infer the past experiences of the sample and compensate for the lack of type or quality of data.

This paper provides three methods for the effective integration of the risk theory and life course theory. Firstly, in terms of research methodology, the life course can be used both as a theoretical framework and as a study method [41], particularly in the analysis of the life event history of risk, and the interaction mechanisms of individual risk trajectories. In trajectories, there is both a “turning point”, i.e., the experience of an event, and a “line”, i.e., the long-term impact that the experience leaves on the individual [42]. Therefore, this paper combines historical public health emergencies with variables such as age and occupation to determine the trajectories of individuals in the public health domain. Secondly, as for the usage of variables, the concept of risk should be considered not only in terms of technical or material risks but also as perceived risks with plenty of complexity. Besides, exploration of the life course element of risk should go beyond the inclusion of simple variables such as age and occupation, and rather find the temporal and trajectory implications of these variables [43]. For example, this paper uses cluster analysis to make age a variable for surveying “whether or not one has experienced a major public health emergency”, while occupation can distinguish the role of individuals in the pandemic and whether or not they are “experiencing the risk currently.” Thirdly, in the analysis of causality, taking a longitudinal perspective will emphasize robust causal mechanisms caused by the life course in the risk domain, such as this paper’s research target: the impact of past major public health emergencies on risk perceptions and protective measures.

### 4.4. Limitations

This paper is theoretically and empirically innovative, but some limitations need to be considered. Firstly, the structural bias of the sample may affect the accuracy of the results, as few people have experienced a major public health emergency. Therefore, the PSM model serves to balance the sample profile, which is also one of the highlights of this paper. Secondly, due to the time required for questionnaire collection, we were unable to obtain findings at an earlier stage of the COVID-19, such as before the full-blown outbreak, which leaves the possibility that later knowledge, perceptions, and behaviors may have interacted in a complex way to affect the study’s accuracy. However, this limitation has little negative influence on the conclusion, because previous studies pointed out that differences in prevention behaviors become smaller over time [5,7], which does not help us to test the hypothesis. Thirdly, the standard life history paradigm requires a high degree of refinement of individual information. As this paper is an initial exploration of the combination of the two methodologies in a public health scenario, it can only temporarily abandon the examination of the role of the other life factors of individuals, pending a subsequent more extensive and in-depth investigation of the data. Finally, in the early stage of the outbreak, China’s quarantine policy greatly hindered the collection of offline questionnaires. Therefore, our questionnaires were mainly collected online, which would bring a certain sample bias, because people who did not use the internet could not participate in the survey.

### 4.5. Conclusions 

This study provides valuable insights for normalizing prevention policies regarding public health emergencies, especially in slacker times of governance. Countless individual public health experiences can shape the collective memory of resistance to diseases. The past can not only provide experience at the government management level but also shape the protective measures at the population level for efficiently handling similar subsequent emergencies [44], with potential to reduce later social and health costs. Our study indicates that it is related closely between post public health emergency experience and proper protective measures towards normalization of risk, so in order to maximize the role of experience, post-public health emergency education is critical. While present public health emergencies may be eventually well under control, future research is warranted to investigate how to utilize public health experiences for early risk governance.

## Figures and Tables

**Table 1:** Measurement of outcomes.

Outcomes	Measurements	Items	Scales
Risk perception	Catastrophic potential: Please judge the seriousness of the following events according to your personal feelings. (R1)	The epidemic situation globally (R10)	Not serious—1 Not too serious—2 Moderately serious—3 Relatively serious—4 Very serious—5
		The epidemic situation in China (R11)	
		The epidemic situation in your province (R12)	
		The epidemic situation in your city (R13)	
		The epidemic situation in your district/county (R14)	
		The epidemic situation in your community/street (R15)	
		The status of your friends’ illness and infection (R16)	
		The status of your families’ illness and infection (R17)	
	Familiarity: Here are some facts about the COVID-19 outbreak. Please select how well you know them. (R2)	The outbreak in China began in Wuhan, Hubei province (R20)	Don’t know at all—1 Little know—2 Roughly know—3 Relatively know—4 Fully know—5
		Cases have been reported in all Chinese provinces (R21)	
		Cases have been found in more than 200 countries and territories around the world (R22)	
		The number of confirmed cases worldwide has exceeded 1.5 million (R23)	
		The obvious symptoms of COVID-19 are high fever and cough (R24)	
		Severe cases have difficulty breathing and even death (R25)	
		The virus is transmitted from person to person (R26)	
		The period of isolation of suspected patients shall not be less than 14 days (R27)	
		You need to wear a mask when going out during the pandemic (R28)	
		Gatherings should be curtailed during the pandemic (R29)	
	Control: Please select how much you agree with the following statements about the COVID-19 epidemic. (R3)	I think the epidemic in China is preventable and controllable (R30)	Completely disagree—1 Disagree—2 Roughly agree—3 Agree—4 Completely agree—5
		I think the extent of the epidemic in this country can be controlled (R31)	
		I think the domestic epidemic will continue to shrink (R32)	
		I think the death toll from the domestic outbreak is manageable (R33)	
		I think the rate of death in the domestic epidemic will continue to slow down (R34)	
	Dread: Please select the extent to which the statement about your personal mood changes during the pandemic matches your real situation. (R4)	During the pandemic, I was afraid of human contact (R40)	Don’t conform at all—1 Little conform—2 Roughly conform—3 Relatively conform—4 Fully conform—5
		During the pandemic, I was afraid of going the public places (R41)	
		During the pandemic, I worried that my previous neglect of protection would cause the disease (R42)	
		My fear of COVID-19 is like my fear of AIDS (R43)	
		I am so scared that I will infect with COVID-19 (R44)	
		I think the symptoms of COVID-19 will make me very miserable (R45)	
		I think I have a chance of dying from COVID-19 (R46)	
		I’m afraid of my family and friends infecting with COVID-19 (R47)	
		Because of COVID-19, the rhythm of my life has been disrupted, which makes me anxious (R48)	
		I’m afraid of being quarantined (R49)	
Individual protective measures	Compared to normal times, do you conduct any following measures during the pandemic? (I1)	Do you wash your hands more often at home? (I10)	No—0 Yes—1
		Do you take hand sanitizer and alcohol with you when you go out? (I11)	
		Do you wear a mask when you go out? (I12)	
		Do you have different forms of disinfection when you go home? (I13)	
		Are you more aware of your body? (I14)	
		Have you ever taken any medicine to prevent a cold? (I15)	
		Have you significantly reduced the number of times you go out? (I16)	
		Have you significantly less reduced the number of using public transportation? (I17)	
		Have you significantly reduced the number of times you go to the mall or supermarket? (I18)	
		Have you significantly reduced the number of get-togethers with your friends and family? (I19)	

**Table 2:** Characteristics of outcomes.

	Items				Total (Standardized)			
Outcomes	Items	*n*	Mean	SD	Mean	SD	Min	Max
Risk perception	R1	1000	2.302	0.520	0	1	−3.924	2.843
	R2	1000	4.369	0.323				
	R3	1000	4.197	0.481				
	R4	1000	3.882	0.623				
Individual protective measures	I1	1000	0.906	0.095	0	1	−5.336	0.986

**Table 3:** Cluster analysis on age based on risk perception and protective measures.

	Mean	SD	Min	Max
Age: 19–46 (*n* = 620)				
Age	33.926	7.672	19	46
Risk perception	0.123	0.943	−3.924	2.496
Protective measures	−0.227	1.016	−5.336	0.986
Age: 47–91 (*n* = 380)				
Age	58.326	6.392	47	91
Risk perception	−0.200	1.057	−3.913	2.843
Protective measures	0.371	0.853	−3.228	0.986

**Table 4:** Characteristics of study participants (*n* = 1000).

	*n*	%
Residence		
City	471	47.1
Town	129	12.9
Suburban area	67	6.7
Rural area	333	33.3
Sex		
Male	508	50.8
Female	492	49.2
Age		
≤30	260	26.0
31–40	200	20.0
41–50	220	22.0
51–60	150	15.0
>60	170	17.0
Education		
Middle school and below	96	9.6
High school or vocational school	243	24.3
University or college	631	63.1
Graduate student or above	30	3.0
Marriage status		
Never married	151	15.1
Married	830	83.0
Divorced or widowed	19	1.9
Family income (RMB/year)		
0–4400	298	29.8
4401–8000	427	42.7
8001–40,000	275	27.5
Minor family members		
Have	653	65.3
Elderly family members		
Have	693	69.3
Family members of chronic patients		
Have	511	51.1
Self-evaluation of health		
Very bad	11	1.1
Bad	31	3.1
Neutral	312	31.2
Good	496	49.6
Very good	150	15.0
Self-evaluation of social status		
Very low	139	13.9
Low	337	33.7
Neutral	415	41.5
High	104	10.4
Very high	5	0.5
Self-evaluation of trust in society		
Very low	10	1.0
Low	47	4.7
Neutral	236	23.6
High	547	54.7
Very high	160	16.0
Self-evaluation of trust in authority		
Very low	5	0.5
Low	16	1.6
Neutral	119	11.9
High	565	56.5
Very high	295	29.5
Having ever witnessed a public health emergency before COVID-19 pandemic		
Yes	380	38.0
Having ever experienced public health emergency before COVID-19 pandemic		
Yes	12	1.2
Currently experiencing public health emergency as of interview		
Yes	158	15.8

**Table 5:** The causal effect of personal experience of public health emergency on risk perception.

	Treated	Controls	ATT	SE	T	Γ
Having ever witnessed public health emergency before COVID-19 pandemic						
Nearest	380	620	−0.200	0.206	0.32	2
Radius	380	620	−0.200 ***	0.082	−3.92	2
Kernel	380	620	−0.200	0.141	−1.07	2
Mahalanobis	380	620	−0.200	0.206	0.32	2
Having ever experienced public health emergency before COVID-19 pandemic						
Nearest	12	988	−0.020	0.406	1.58	>2
Radius	10	988	−0.020	2.637	−0.01	>2
Kernel	12	988	0.020	0.404	0.68	>2
Mahalanobis	12	988	−0.020	0.406	1.58	>2
Currently experiencing public health emergency as of interview						
Nearest	158	842	−0.042 **	0.129	−2.15	>2
Radius	158	842	−0.042	0.200	−0.25	>2
Kernel	157	842	−0.041	0.098	−0.78	>2
Mahalanobis	158	842	−0.042 **	0.129	−2.19	>2

Significance: *** *p* < 0.001, ** *p* < 0.01, Abbreviation: ATT (the average effect of treatment in the treated individuals); SE (standard errors in parentheses); T (*t*-test).

**Table 6:** The causal effect of personal experience of public health emergency on protective behavior.

	Treated	Controls	ATT	SE	T	Γ
Having ever witnessed public health emergency before COVID-19 pandemic						
Nearest	380	620	0.371 ***	0.198	5.88	>2
Radius	380	620	0.371 ***	0.080	7.50	>2
Kernel	380	620	0.371 ***	0.147	4.10	>2
Mahalanobis	380	620	0.371 ***	0.198	5.88	>2
Having ever experienced public health emergency before COVID-19 pandemic						
Nearest	12	988	0.898 **	0.261	2.02	>2
Radius	12	988	0.898	2.622	0.35	>2
Kernel	10	988	0.881 ***	0.179	4.51	>2
Mahalanobis	12	988	0.898 **	0.261	2.02	>2
Currently experiencing public health emergency as of interview						
Nearest	158	842	0.319 *	0.118	1.81	>2
Radius	158	842	0.319 *	0.198	1.91	>2
Kernel	157	842	0.315 **	0.089	2.04	>2
Mahalanobis	158	842	0.319 *	0.118	1.81	>2

Significance: *** *p* < 0.001, ** *p* < 0.01, * *p* < 0.05. Abbreviation: ATT (the average effect of treatment in the treated individuals); SE (standard errors in parentheses); T (*t*-test).

**Table 7:** The causal effect of risk perception on protective behavior under different personal experience of public health emergency.

	Overall	Having Ever Witnessed		Having Ever Experienced		Currently Experiencing	
		Yes	No	Yes	No	Yes	No
	(*n* = 1000)	(*n* = 380)	(*n* = 620)	(*n* = 12)	(*n* = 988)	(*n* = 158)	(*n* = 842)
Risk perception	0.179 ***	0.135 *	0.210 ***	0.137	0.165 ***	−0.020	0.206 ***
	(0.030)	(0.041)	(0.042)	(0.337)	(0.031)	(0.071)	(0.033)
Witnessed	−0.428 ***						
	(0.084)						
Experienced	0.519						
	(0.272)						
Experiencing	0.271 ***						
	(0.082)						

Significance: *** *p* < 0.001, * *p* < 0.05. Standard errors in parentheses.

## Data Availability

The data that support the findings of this study are available from the corresponding author upon reasonable request.
